# Assessing karyotype precision by microarray-based comparative genomic hybridization in the myelodysplastic/myeloproliferative syndromes

**DOI:** 10.1186/1755-8166-3-23

**Published:** 2010-11-15

**Authors:** Marilyn L Slovak, David D Smith, Victoria Bedell, Ya-Hsuan Hsu, Margaret O'Donnell, Stephen J Forman, Karl Gaal, Lisa McDaniel, Roger Schultz, Blake C Ballif, Lisa G Shaffer

**Affiliations:** 1 Department of Cytogenetics, City of Hope, Duarte, CA, USA; 2 Department of Biostatistics, City of Hope, Duarte, CA, USA; 3 Department of Hematology/Hematopoietic Cell Transplantation, City of Hope, Duarte, CA, USA; 4 Department of Pathology, City of Hope, Duarte, CA, USA; 5 Signature Genomics, Spokane, WA, USA; 6 Quest Diagnostics Nichols Institute, Chantilly, VA, USA

## Abstract

**Background:**

Recent genome-wide microarray-based research investigations have revealed a high frequency of submicroscopic copy number alterations (CNAs) in the myelodysplastic syndromes (MDS), suggesting microarray-based comparative genomic hybridization (aCGH) has the potential to detect new clinically relevant genomic markers in a diagnostic laboratory.

**Results:**

We performed an exploratory study on 30 cases of MDS, myeloproliferative neoplasia (MPN) or evolving acute myeloid leukemia (AML) (% bone marrow blasts ≤ 30%, range 0-30%, median, 8%) by aCGH, using a genome-wide bacterial artificial chromosome (BAC) microarray. The sample data were compared to corresponding cytogenetics, fluorescence *in situ *hybridization (FISH), and clinical-pathological findings. Previously unidentified imbalances, in particular those considered submicroscopic aberrations (< 10 Mb), were confirmed by FISH analysis. CNAs identified by aCGH were concordant with the cytogenetic/FISH results in 25/30 (83%) of the samples tested. aCGH revealed new CNAs in 14/30 (47%) patients, including 28 submicroscopic or hidden aberrations verified by FISH studies. Cryptic 344-kb *RUNX1 *deletions were found in three patients at time of AML transformation. Other hidden CNAs involved 3q26.2/EVI1, 5q22/APC, 5q32/TCERG1,12p13.1/EMP1, 12q21.3/KITLG, and 17q11.2/NF1. Gains of CCND2/12p13.32 were detected in two patients. aCGH failed to detect a balanced translocation (n = 1) and low-level clonality (n = 4) in five karyotypically aberrant samples, revealing clinically important assay limitations.

**Conclusions:**

The detection of previously known and unknown genomic alterations suggests that aCGH has considerable promise for identification of both recurring microscopic and submicroscopic genomic imbalances that contribute to myeloid disease pathogenesis and progression. These findings suggest that development of higher-resolution microarray platforms could improve karyotyping in clinical practice.

## Introduction

The myelodysplastic syndromes (MDS) comprise a heterogeneous group of clonal hematopoietic cell disorders characterized by ineffective hematopoiesis and a highly variable clinical course, ranging from indolence over many years to rapid progression to acute myeloid leukemia (AML). MDS is also closely related to the World Health Organization (WHO) classification entities of MDS/myeloproliferative neoplasia (MPN) and AML with myelodysplasia-related changes [1]. Because the features of MDS are heterogenous and the majority of MDS patients are ≥ 60 years old [2], major research efforts have focused on identifying new biological and prognostic markers to optimize and detoxicify therapy for myeloid neoplasias [3-8].

The International Prognostic Scoring System (IPSS) was introduced in 1997 for evaluation of primary MDS patients to predict overall survival and leukemia-free survival [9]. This "gold standard" scoring system is based on three key prognostic factors: the number of peripheral blood cytopenias, percentage of bone marrow blasts, and cytogenetics. Although cytogenetics is one of the most valuable diagnostic and prognostic indicators in MDS, a limiting factor of the IPSS cytogenetics score is that only 50% of primary MDS patients show recurring cytogenetic aberrations [10], underscoring the need to improve the resolution of cytogenetic technology. Approximately 90% of the karyotypic changes observed in MDS are unbalanced chromosome aberrations leading to gains or losses in all, or part of, specific chromosomes [1], with the most common karyotypic aberrations incorporated into the IPSS. Recent clinical trials report MDS patients with specific cytogenetic aberrations show improved efficacy with targeted therapy; for example, del(5q) low- and intermediate-risk MDS patients show high responsiveness to the immunomodulatory drug lenalidomide [11,12], and monosomy 7 MDS patients show high responsiveness to demethylating agents [13,14]. With the emergence of new targeted therapeutic alternatives, current MDS cytogenetic investigations are focusing on the need to increase sensitivity and resolution of karyotyping technology to uncover novel cytogenetic defects, and to correlate these findings with targeted biological activity, response to therapy and clinical outcome [5-8,15].

Recent translational research studies have shown that genome-wide microarray testing is a powerful technology for detecting recurring, submicroscopic alterations in genes that contribute to the pathogenesis of MDS [6,8,16-19]. Such encouraging data suggest that higher-resolution chromosomal microarray testing will improve our diagnostic and prognostic potential in MDS; however, before implementation in diagnostic laboratories, extensive evaluation of the technology (assays) must be initiated to define clinical utility, sensitivity, reproducibility and data analysis/interpretation limitations. As a first step, we initiated an exploratory study using a genome-wide bacterial artificial chromosome (BAC)-based microarray to determine if known cytogenetic imbalances and potentially important submicroscopic cytogenetic aberrations could be detected by microarray-based comparative genomic hybridization (aCGH) in MDS/MPD samples collected during routine clinical evaluation. The aCGH results were compared to their corresponding conventional cytogenetics, fluorescence *in situ *hybridization (FISH), and clinical-pathological findings. Newly defined deletions and gains were validated by FISH analysis.

## Materials and methods

### Patient Samples

Upon institutional review board approval (IRB# 07245, 04187, 95124), we queried the City of Hope cytogenetic database to identify patients with clinical indications of MDS, who had residual material available for study. A total of 37 bone marrow samples from 30 patients collected between 2005 and 2008 were eligible for study. Follow-up studies were evaluated in five patients to evaluate assay reproducibility and clonal evolution of disease detection. When available, the bone marrow aspirate and biopsies were reviewed to confirm a diagnosis of MDS based on the French-American-British (FAB) and WHO classifications [20,21], bone marrow cellularity, the peripheral blood parameters, and lineage dysplasia (Table 1). During the pathology re-inspection of this retrospective analysis, seven patients were re-classified as MDS in transformation to AML because the bone marrow blast percentage was between 20-31%, exceeding the WHO pathological diagnosis of MDS [21] but within the FAB MDS classification [20]. Two patients were diagnosed with MPN or MPN/AML: patient 29 was diagnosed with primary myelofibrosis, and patient 21 was diagnosed with AML arising from MPN (JAK2 mutation positive polycythemia vera) with 25% blasts. One RAEB-2 patient (#27) showed a normal karyotype; the remaining 29 patients showed karyotypic findings consistent with a myeloid disorder. Patient #30 showed a der(1;7)(q10;p10) after culturing in a medium supplemented with myeloid growth factors [22], quantitated by FISH analysis as >5% in a direct preparation. The low-level der(1;7) clone was present and stable in this patient for five years with no pathological evidence of disease.

**Table 1:** Clinical, Cytogenetics and Pathological Data

Number of patients	30
Gender (M/F)	18/12

Age at study, range (median)	19-87 (64 yr)

Peripheral blood values (n = 29)	

WBC* (K/CMM) range (median)	1.2 - 45.5 (3.1)

Hb* (g/dl) range (median)	7.4-15.6 (9.8)

Platelet* (K/CMM) range (median)	14 - 598 (82)

Eosinophils* % range (median)	0-9.2 (1.3)

Bone marrow blasts, range (median)	0-31% (8.0%)

Cellularity	

Hypercellular	17

Normocellular	7

Hypocellular	4

Fibrotic or aparticulate	2

FAB^# ^	

RA	1

RARS	2

RAEB	15

RAEB-t	5

Refractory cytopenia, NOS	2

Hypoplastic MDS	1
MDS, NOS	1*
MPN → AML	1
Idiopathic myelofibrosis	1

Abnormal cytogenetics	1

WHO	

5q-syndrome	1

RARS	1

RCMD	2

RAEB-1	2

RAEB-2	8

AML	3

t-MDS	8

t-AML	2**

Other pathology	

AA/hypoplastic MDS	1

Primary myelofibrosis	1

Cytogenetic abn only^	1

IPSS score - for 16 patients	

HIGH	2

INT-2	10

INT-1	2

LOW	2

History of previous chemo- or radiotherapy	14^

# Samples were submitted with a working diagnosis of MDS. Pathology review revealed the myeloid diagnoses presented in this table. *Aspirate was aparticulate and hemodilute (insufficient for pathology review). **Blast percentage between 22-31% when sample underwent morphologic evaluation. ^Patients were previously treated for AML (4), MDS (1), Hodgkin lymphoma (3), non-lymphoma (3), breast cancer (1), prostate (1), and prostate/kidney cancer (1).

The aCGH results were compared to the patients' corresponding clinical, cytogenetic and pathological characteristics. The medical records were reviewed to confirm the clinical diagnoses and associated demographics, including age, sex, race, IPSS score, previous history, treatment status, and transformation to AML.

### Cytogenetics and FISH validation studies

Cytogenetic and FISH studies were performed using standard methods. The cytogenetics results were reviewed to confirm the karyotypic diagnosis, number of secondary karyotypic changes, and overall karyotype complexity. With the exception of patient #17, at least 20 mitotic cells were analyzed and the non-random cytogenetic aberrations were described according to ISCN 2009 [23]. Many of the aberrations observed by conventional cytogenetics were confirmed by FISH studies for follow-up minimal residual disease (MRD) testing using standard methods. Locus-specific FISH analyses were performed to confirm the recurring hidden CNAs detected by aCGH. For the previously unidentified imbalances or "cryptic" aberrations (≤ 10 Mb), the probes used for FISH analysis were carefully chosen and mapped within the genomic coordinates of the specific chromosome region showing gain or loss. In each case, a control DNA FISH probe from the opposite chromosome arm was included to verify the CNAs in relation to cell ploidy. The following probes were obtained from Abbott Molecular, Inc (Des Plaines, IL): D5S23/D5S721 (5p15.2), EGR1 (5q31), CEP7/pZ7.5, D7S486 (7q31), D8Z2 (CEP8), MYC (8q24.12-q24.13), CDKN2A (9p21), CEP9 (9p11-q11), MLL (11q23), ETV6 (12p13), CEP15 (D15Z4), RARA (17q12), D20S108 (20q12), and RUNX1 (21q22). Homebrew probes were used to confirm other CNAs: RP11-96F24 (1p13.3), RP11-104L21 (1q24.2), RP11-892D8 (HDAC4/2q37.3), RP11-107C15 (APC/5q22), RP11-134O21 (8p23.2), RP11-103I15 (11q14.3), RP11-47N15 (NCAM/11q23.1), RP11-1069E18 (EMP1/12p13.1), RP11-34A16 (ETV6/12p13.2), RP11-928N17 (CCND2/12p13.32), RP11-1023C8 (PDE3A/12p12.2), RP11-92E19 (HEBP1/12p13.1), RP11-147E12 (12p12.3), RP11-978A23 (SP1/12q13.1), RP11-806H9 (KITLG/DUSP6/12q21.3), RP11-353O18 (RB1/13q14.3), RP11-199F11 (TP53/17p13.1), RP11-353O18 (NF1/17q11.2), RP11-62N23 (ERBB2/17q21.1), RP11-300O12 (MAPT/17q21.31), RP11-838N2 (TGIF/18p11.31), RP11-467I15 (18q23), RP11-110K14 (SLC24A3/20p11.23), and for RUNX1 (21q22) RP11-77G18 or BAC dJ1107L6, the latter kindly provided by Dr. Mario Rocchi (Bari, Italy).

Two hundred cells were scored for interphase FISH (I-FISH). When applicable, 3-5 metaphase cells were reviewed for chromosomal localization of the CNAs as previously described [24]. The FISH slides were scanned, localized and recorded on the slide using the BioView Duet image analyzer (BioView, Ltd., Rehovot, Israel).

### Microarray analyses

DNA was isolated from frozen buffy coat specimens using the EZ1 tissue kit and robot (Qiagen, Inc. Valencia, CA) per manufacturer's protocol. No myeloid cell enrichment techniques were employed. After isolation, DNA concentrations and quality were evaluated by spectrophotometry using the NanoDrop ND-1000 (NanoDrop Technologies, Wilmington, DE) and by agarose-gel electrophoresis. To evaluate array reproducibility, three independently collected non-remission samples from a single patient, two samples (right and left iliac crest) collected from a single patient on the same day, and five follow-up non-remission samples from different patients were evaluated. All samples were processed using the SignatureChipWG™ BAC microarray (v1.0.1) following previously published methods [25]. The nucleotide positions listed in SignatureChipWG v1.0.1 are based on the UCSC Genome Browser's March 2006 human reference sequence (hg18; NCBI Build 36.1). The aCGH results were described according to ISCN 2009 [23]. Normal (non-pathogenic) copy number variants (CNV) were not included in the aCGH results (Database of Genomic Variants, http://projects.tcag.ca/variation/).

### Statistical analysis

The demographic and clinical covariates were compared using ANOVA for continuous variables and Fisher's exact test for categorical responses.

## Results

### Characterization of study cohort

The clinical-pathological characteristics of the patients studied are listed in Table 1. The median age was 64 years (range 19 - 87) with 18 males and 12 females. The pathology and cytogenetics evaluation verified that 29 of 30 patients had a myeloid malignancy and 29 patients had one or more karyotypic aberrations commonly associated with a myeloid disorder. All samples analyzed by aCGH had a bone marrow blast percentage of ≤ 31% (range 0-31%, median 8%). Nine patients showed complex karyotypes (≥ 3 clonal aberrations), 20 patients showed simple karyotypes (one to two clonal aberrations), and one RAEB-2 patient showed a normal karyotype. IPSS scores were calculated for 16 patients: 12 patients had INT-2/High scores and four patients had INT-1/Low scores. Fourteen patients had a history of radiation or chemotherapy exposure and were categorized with therapy-related disease.

### Validation of microarray assay reproducibility

Reproducibility of the microarray assay was determined in two separate studies. First, DNA was extracted and processed independently from both the left and right iliac crest from patient #9. The aCGH profiles from both samples showed remarkable agreement with the corresponding complex karyotype. I-FISH confirmed the presence of new or hidden aberrations detected by aCGH. A follow-up sample from this patient with active disease also confirmed the recurring CNAs. Secondly, three samples collected from a single patient (#19) at different time points were evaluated. The three samples showed varying levels of residual disease (30%~70%) characterized by monosomy 7 and an interstitial ~11 Mb deletion of the long arm of chromosome 12. Monosomy 7 was evident by conventional cytogenetics and confirmed by I-FISH, whereas the 12q deletion uncovered by aCGH become apparent during re-inspection of the longer chromosomes 12 in the patient's karyograms. All three aCGH studies of patient #19 showed excellent reproducibility of the BAC microarray calls.

### Comparison of cytogenetic, FISH and aCGH results

Table 2 lists the cytogenetic, FISH and aCGH results for the first sample analyzed for all 30 patients. aCGH detected CNAs in 25 (83%) of 30 MDS/MPN patient samples. Abnormalities were detected on all chromosomes except chromosomes 10, 14, 16, × and Y. The range of number of CNAs per patient was 0 to 23 (median 2.0 CNAs/patient) with losses more common than gains. Thirteen patients showed one to two CNAs, 11 patients showed ≥ 3 CNAs and six patients showed no pathological CNAs (including one karyotypically normal patient). Unexpected chromosome instability was observed in five chromosome arms (5q, 6p, 7q, 12p, 19p) which showed multiple or non-contiguous CNAs on the aCGH chromosome plots. Owing to non-contiguous gains and losses, a total of 21 chromosome 7 deletions in 16 patients and 16 5q deletions in 12 patients were observed. The 5q deletions were highly variable with eight different proximal breakpoints and nine different distal breakpoints. Chromosome 12 showed 17 CNAs with a nearly equal number of gains (n = 7) and losses (n = 9) in the short arm. Two patients showed surprisingly complex 12p aCGH plots, with multiple losses and gains, including FISH-confirmed CCND2 amplification in one patient.

**Table 2:** Cytogenetic, aCGH and FISH Results.

UPN	WHO	AML (Y/N)	Karyotype	aCGH Results by ISCN (2009)	FISH sites/type (percentage) - (size)
**1**	RAEB-2	Y	43~44,XY,-5,der(5)add(5)(p15.3) del(5)(q34),der(7)dup(?7)(p15p22) t(7;15)(q31;q11.2),add(12)(p13), der(15)t(7;15), add(17)(p13),-18, der(19)t(?5;19)(p15.1;p13.3),+mar1[cp2/ 45~46, sl,-Y,-mar1,+mar2x2 [cp3]/ 42~46,sl,r(12) (p11.2q24.3),-der(15) t(7;15),-mar1[8]/ 42-46,sl, dic(6;12) (p10;q10),add(12)(p13),add(17)(p11.2), +add(18)(q23),add(20) (p13),-mar1[cp3]/42~45, sdl3,psu dic (18;19)(p11.2;p13.3)t(?5;19) (p15.1;p13.3)[cp4]	arr 5p15.33p15.31(387,034-7,150,950)x1,5q22.2(112,073,070-112,236,540)x1, 5q23.1q35.3(119,285,451-180,616,147)x1,6p25.3p25.2(326,849-2,927,819)x1, 7p14.1(40,423,354-42,301,602)x1,7p13q11.22(44,583,533-67,706,469)x1, 7q11.22(69,795,761-71,039,199)x1,7q21.3(94,315,008-94,637,930)x1,7q21.3 q36.3(96,419,102-158,788,150)x1,11q14.1q25(84,811,642-34,301,424)x3~4, 12p13.33(74,345-1,781,320)x3,12p13.32p13.31(4,144,817-6,740,664)x3, 12p13.31(7,454,167-7,781,482)x1,12p13.31(7,909,595-8,174,285)x3,12p13.2 (10,290,689-10,555,515)x1,12p12.3 p12.2(16,484,810-20,837,343)x1,12p12.1 (23,481,114-23,903,637)x3,12p11.22p11.1(27,792,939-33,636,183)x1,17q11.2 (26,415,260-26,685,081)x1,18p11.32p11.22 (905,705-10,600,909)x1,18q21.32 q23(55,877,565-76,103,395)x1,19p13.3(211,754-719,804)x1,20p11.23 (19,446,369-19,632,379)x1	5q22.2/APC loss (73%) (164 kb) 5q23.1q35.5 loss (69.2%) (61.3 Mb) 11q14.1q25 gain (42%) (49.5 Mb) 12p13.32/CCND2 gain (67%) (2.6 Mb) 12p12.3p12.2 loss (79%) (4.3 Mb) 12p12.1/SOX5 gain (23%) (422 kb) 17q11.2/NF1 loss (33%) (270kb) 18p11.32p11.22 loss (21%) (9.6 Mb) 18q21.32q23 loss (48%) (20.2 Mb) 20p11.23/SLC24A3 loss (50%) (186 kb)

**2**	t-MDS (RAEB-1)	N	45,XY,-7,add(18)(p11.2) [20]	arr 7p22.3q36.3(106,470-158,788,150)x1,9p24.3p13.1(188,707-38,662,411)x3, 18p11.32p11.21(140,284-14,065,199)x1	Monosomy 7 (80%) (158 Mb) aCGH defined der(18)t(9;18)(p13.1; p11.32)

**3**	RCUD	N	40,X,-Y,der(5;12)(q10;q10),-7,-13, der (?5;15)(p10;q10),der(17;?21) (q10;q10) ,-18,-22,+mar[3]/80,slx2 [3]/46,XY[14]	arr 5q32q33.1(145,784,050-150,381,359)x1~2,7p22.3q36.3(106,470-158,615,766) x1~2,13q14.12 q33.3(45,226,907-108,905,138)x1~2,18p11.32q23(140,284-76,103,395)x1~2	FISH non-informative: 2n/4n clones present. Loss of Y not detected by aCGH.

**4**	RAEB-2	N	46,XX,der(1)t(1;5)(p13;q13),-4,del(5) (q13),+der(?)t(1;?) (p13;?)[9]/ 46,XX[11]	arr(1-22,X)x2 Normal Female	5q deletion (0.7%) (within background) Suspect low-level clonality

**5**	t-MDS (RAEB-1)	UNK	46,XY,t(3;12)(p25;q13)[16]/46,XY[4]	arr(1-22)x2,(XY)x1 Normal Male	Balanced translocation

**6**	t-MDS (RAEB-1)	UNK	46,XY,del(5)(q15q31)[13]/46,XY[6]	arr 5q14.1q33.3(80,739,035-157,165,622)x1	del(5q) (17.5%) (76.4 Mb)

**7**	RAEB-2	Y	46,XX,t(2;6)(q33;p11.2),r(11)(p15q23)[18]/46,idem,del(X)(q24q28)[2]	arr 2q33.1q37.3(202,851,669-242,436,891)x1,6p22.2p22.1(25,845,975-26,237,877)x1,6p12.3(47,118,924-47,484,069)x1,11p13(32,426,278-33,841,461)x1,11p13q14.1(35,917,783-79,934,744)x3,11q14.1q25(84,987,721-134,431,368)x1	2q33.1q37.3 deletion (87%) (39.6 Mb)

**8**	RCMD	N	46,XX,+1,der(1;7)(q10;p10)[20]	arr 1q21.1-1q44(1444111146-247189904)x3,7q11.21-7q36.3(61991850-158615766)x1,?13q21.1-13q21.33(56,970,230 -71,102,396)x1~2	del(7q) (48.5%) (96.6 Mb) suspected 13q deletion - unable to confirm

**9**	t-MDS (RAEB-2)	Y	43~47,XX,+2[2],del(3)(q23q29)[3],-4[4], add(4)(p14)[3],del(5)(q13)[17],del(6) (q23q27)[3],del(7)(q21.2q36)[17], +8[2],+9[2],+11[3],add(11)(p15)[16], add(12)(p11.2)[2],-13[13],+14[2],-17[7],-18[9],-20[5],del(20)(q11.2q13.3)[2], der(21)t(9;21)(q22;q22)[15],+mar1[6], +mar2[12],+mar3[2][cp17]/46,XX[3]	arr 3q22.2q29(136,891,829-199,230,435)x3,5q14.1q34(80,739,035-162,985,861) x1,6p25.3p22.1(89,702-27,735,846)x1,6p21.33p21.1(31,007,155-44,123,999)x3, 7q21.3(94,315,008-94,637,930)x3,7q21.3q36.3(95,908,715-155,963,689)x1, 8q24.13q24.3(123,825,412-145,957,473)x3,11p15.5p15.4(896,316-7,391,465)x1, 11q23.1q25(111,095,142-134,431,368)x3,12q13.13(50,423,182-52,714,396)x3, 13q12.11q12.3(18,448,674-30,094,861)x3,13q13.1q34(31,498,180-114,103,243) x1,17q21.31(41,288,422-41,528,254)x3,18p11.32p11.22(140,284-10,487,828)x1, 18q21.2q23(51,018,278-76,103,395)x1,20q11.23q12(35,843,902-39,677,519)x1, 20q13.2(52,310,955-52,627,305)x1,21q22.2q22.3(39,838,925-45,584,697)x1, 22q11.22q11.23(21,330,008-21,978,854)x3	12q13.13/SP1 gain (25%) (2.3 Mb) 17q21.31/MAPT gain (22.5%) (240 kb) Array reproducibility confirmed same CNAs detected on right and left PIC aspirates. Follow-up study confirmed 5q, 7q and most deletions.

**10**	RAEB-1	N	46,XY,del(20)(q11.2q13.3)[2]/46,XY[18]	arr(1-22)x2,(XY)x1 Normal Male	20q deletion (2%) Low-level clonality

**11**	RAEB-2	N	46,XX,del(5)(q13q33)[10]/46,sl, del(11)(q13q23)[8]/46,XX[2]	arr 5q14.3q34(87,963,057-162,985,861)x1,11q14.1q23.1(79,505,241-111,400,572)x1~2	5q deletion (38.4%) (75 Mb) MLL FISH (0.4%) (WNL)

**12**	Relapsed AML (RAEB-T)	Y	46,XY,i(7)(p10)[17]/46,XY[3]	arr 7p22.3q11.21(106,470-62,303,249)x3,7q11.22q36.3(67,538,481-158,788,150)x1	del(7q) (60.0%) (91 Mb)

**13**	RARS	N	46,XY,del(5)(q15q31)[20]	arr 5q21.1q32(98,042,952-145,952,287)x1	del(5q) (70.7%) (47.9 Mb)

**14**	RAEB-2	Y	46,XY,del(11)(q13q25)[20]	arr 11q14.1q25(84,811,642-132,230,180)x1	

**15**	fibrotic MDS (RAEB-1)	UNK	44,XY,add(5)(q13),del(9)(p10),der(11;15)(q10;q10),der(13)t(7;13;?) (13pter- > 13q22 : :7q31- > 7q32::?), der(18)t ?11;18)(p11.2;q23),psu dic(20;7) (:7p11.2->7q11.2::20p13- > 20qter)[24]	arr 5q21.1q35.3(102,768,888-180,616,147)x1,7p22.3p11.1(106,470-7,622,921)x1, 7q11.21q22.3(61,991,850-107,152,110)x1,7q31.33q32.3(126,838,584-130,116,345)x1,7q36.1q36.3(148,606,208-158,788,150)x1,12p13.32p13.31 (4,144,817-8,174,285)x3,2p13.2p12.3(10,290,689-16,828,705)x1,12p12.2 p12.1(20,350,473-23,903,637)x3,13q21.1q21.2(57,084,770-59,940,562)x1, 18q22.1q23(62,148,705-76,103,395)x1	del(5q) loss (70%) (77.8 Mb) chr7 CEP7/D7S486 (7q31): normal CDKN2A/2B 9p21 loss (44.5%) 12p13.32p13.31/CCND2 gain (49.3%) (4.0 Mb) 12p13.2-12p12.3/EMP1 loss (75.0%) (6.5 Mb) 1212p12.2-12p12.1/SOX5 gain (54.5%) (3.5 Mb)

**16**	RAEB2 -> AML	Y	45,X,-Y,del(4)(q12q21),-5,add(7)(q11.2), ?t(7;21;12) (q22;q22;p12),?del(17)(p13), +mar[1]/ 45,sl,add(19)(q13.3)[6]/ 90,sdl1x2[1]/46,XY[12]	arr 4p15.31p14(20,201,929-38,418,177)x1,4q13.1q21.23(59,479,326-85,831,534)x1,4q22.1q27(88,240,778-121,956,556)x1,5p13.2p12(38,353,657-42,684,423)x1,5q14.1q21.1(76,836,011-98,453,706)x1,5q23.3q35.3(127,839,105- 180,616,147)x1,7q22.2q36.3(105,379,216-155,347,034)x1,12p13.2p11.1 (10,290,689-33,636,183)x1,17p13.3p11.2(0-17,929,998)x1,19p13.3(211,754-383,987)x1,19p13.3(2,139,294-2,922,392)x3,19p13.3p13.2(5,893,471-7,959,704)x3,19p13.2p13.13(9,747,145-13,087,968)x1,19p13.12p13.11 (14,852,729-19,194,051)x3,21q11.2q22.2(14,429,720-40,367,306)x3	del(5q) (54.3% 2n/4.9% 4n) del(7q) (50.7% 2n/5.5% 4n) 19p13.2/MAP2K7 gain (42.6% 2n/3.5% 4n) (2.1 Mb) 19p13.12p13.11/MAP1 S gain (44%) (4.3 Mb)

**17**	RAEB-2	N	47,XX,+9,del(20)(q11.2q13.1)[2]/46,XX[4] Limited Study	arr 9p24.3q34.3(188,707-140,168,105)x3,20q11.23 q13.12(35,843,902-45,321,690)x1	trisomy 9 (16.5%) del(20q) (10.2%)

**18**	t-MDS (RAEB-1)	N	46,XX,der(7)del(7)(p10)del(7)(q22)[13]/ 45,XX-7[6]/46,XX[1]	arr 7p22.3p11.2(106,470-55,599,166)x1,7q21.3q36.3(94,315,008-158,593,771)x1	del(7q)/ monosomy 7 (84.5%)

**19**	t-MDS (RAEB-1)	N	45,XY,-7[11]/46,XY[9]	arr 7p22.3q36.3(106,470-158,593,771)x1,12q21.31q23.1(84,097,500-95,218,964)x1	monosomy 7 (70%) del(12)q21.3q23 (75%) (11 Mb)
**20**	RAEB-2 and Multiple myeloma	Y	46,XY,del(5)(q15q33)[21]	arr 5q21.1q33.2(98,272,436-153,873,892)x1	del(5q)/-5 (80.5%) trisomy 5 (1.0%) by non-targeted FISH. Plasma cell specific FISH: 30/30 cells = 100.0% Trisomy 5

**21**	AML arising from MPD	Y	47,XX,+8[20]	arr 1p21.3p12(96,795,246-119,008,810)x2~3,8p23.3q24.3(345,060-146,236,298)x3,21q22.12(35,028,342-35,371,865)x1~2	trisomy 8 (82.5%) 1p21.3p12 dup (32%) (22 Mb) RUNX1 deletion (10%) (344 kb)

**22**	t-MDS → AML	Y	46,XX,del(7)(q22q32)[20]	arr 3q26.1q29(166,401,814-199,230,435)x3,7q21.3q36.3(94,315,008-158,788,150)x1,21q22.12(35,028,342-35,371,865)x1~2	del(7q) (91.5%) (64.5 Mb) 3q gain with EVI1 break (94%) (32.8 Mb) RUNX1 deletion (5.0%) (344 kb)

**23**	hypoplastic MDS	N	45,XX,-7,del(12)(p11.2p13)[20]	arr 7p22.3q36.3(106,470-158,788,150)x1,12p13.1p11.1(12,890,018-33,636,183)x1	Monosomy 7 (39.7%) (158.7 Mb) 12p12.1/KRAS loss (58.5%) (20.7 Mb) ETV6/RUNX1 FISH: normal

**24**	5q- syndrome	N	46,XX,del(5)(q11.2q31)[19]/ 46,XX[1]	arr 5q14.3q33.3(87,963,057-158,280,854)x1,12p13.1p12.2(12,961,431-20,567,792)x1~2	del(5q) (65.5%) (70.3 Mb) 12p13.1p12.2/EMP1 deletion (17%) (7.6 Mb)

**25**	t-MDS	N	45,XY,-5,add(17)(p11.2),-18,+mar[6]/ 46,XY[17]	arr(1-22)x2,(XY)x1 Normal Male	TP53 deletion (5.0%)

**26**	t-AML RAEB-T	Y	47,XX,+8[5]/46,XX[15]	arr 8p23.3q24.3(345,060-146,236,298)x2~3,21q22.12(35,028,342-35,371,865)x1~2	Trisomy 8 (22.5%) RUNX1 loss (1.0% or WNL) RP11-77g18 (344 kb)

**27**	RAEB-2	Y	46,XY[20]	arr(1-22)x2,(XY)x1 Normal Male	8q24.3/CTD-3034E3: normal (CNV)

**28**	t-MDS (RCMD-RS)	N	46,XY,del(3)(p21),der(5;15)(p10;q10), der(6)del(6)(p11.2p21.1)del(6) (p23p25), ?del(16)(q22q24),-17,+ider(?),+r [19]/ 46,XY[1]	arr 3p26.3p14.1(46,141-71,438,751)x1,5q15q35.3(92,810,609-180,616,147)x1, 6p25.3p22.1(89,702-27,761,655)x1,6p21.33p21.1(31,007,155-44,123,999)x3, 6p12.3p11.2(45,390,156-58,131,862)x1,15q11.2q13.1(22,577,151-26,079,398) x1,17p13.3p13.1(0-8,045,204)x1	del(5q) (73.4%) 16q22/CBFB FISH: normal TP53 deletion (71.7%)

**29**	Primary myelo-fibrosis	Y	46,XY,+1,der(1;7)(q10;p10)[20]/ 46,XY[1]	arr 1q21.1q44(144,111,146-246,864,638)x3,5q32(145,643,075-145,952,287)x1, 7q11.21q36.3(61,991,850-158,615,766)x1,12p13.1(12,890,018-13,268,329)x1~2, 13q14.2q14.3(47,759,453-49,406,099)x1,17q11.2(26,415,260-27,249,359)x1	5q32/TCERG1 (77%) (309 kb) 12p13.1/EMP1 deletion (6.0%) (378 kb) 13q14.2q14.3/RB(91%) (1.65 Mb) 17q11.2/NF1 deletion (75%) (834 kb)

**30**	normal	N	46,XX,+1,der(1;7)(q10;p10)[5]/ 46,XX[15]	arr(1-22,X)x2 Normal Female	del(7q) (3.0%) and 3p12.1 deletion (70%) (CNV)

UPN - unidentified patient number; WNL - within normal limits; CNV - copy number variation

Excellent agreement between the conventional cytogenetics/FISH and aCGH results were observed in the majority of patients, with aCGH providing more precise breakpoint definition. aCGH confirmed and refined heterogeneous breakpoints in 5q (#6, #11, #13), 11q (#11, #14), 12p (#23), and chromosome 7 (#12, #18) aberrations. In patient #18, the breakpoints for both the p and q arms of the small centromeric-containing fragment, revealed to be chromosome 7 (84.5%) by I-FISH, were defined. The 5q breakpoints were modified from 5q15q31 to 5q21.3q32 in a RARS patient (#13), confirming that RPS14 was not deleted. Other notable findings included the lack of an MLL aberration in patient #11 with a del(11)(q13q23) and lack of an ETV6 deletion in a t-MDS, patient #23, with a del(12)(p11.2p13). In the latter patient, aCGH mapped the 12p deletion to 12p13.1p11.1, a finding verified by I-FISH (58.5%) using a KRAS probe (RP11-34A16).

In addition to refining breakpoints, the increased resolution of the array identified origin of unknown markers, composition of "add" or additions to chromosomes, amplified regions, and unsuspected complex rearrangements within a single cytogenetically defined aberration. In t-MDS patient #2, aCGH revealed the add(18) was a der(18)t(9;18), which changed the karyotype to 45, XY,-7,+9, der(9;18)(p10;q10). Similarly, the suspected balanced t(2;6)(q33;p11.2) and r(11)(p15q23) aberrations in a RAEB-2 patient (#7) were considerably more complex by aCGH, which detected two small deletions within 6p (392-kb deletion at 6p22.2p22.1 and another 365-kb deletion at 6p12.3), a 2q33.1q37.3 deletion, and a complex deletion/duplication/deletion ring chromosome 11 resulting in three derivative chromosomes. However, the sideline clone, composed of only two mitotic cells (10%) with a small interstitial deletion of Xq24q28, was not detected by BAC aCGH.

**Figure 1:**
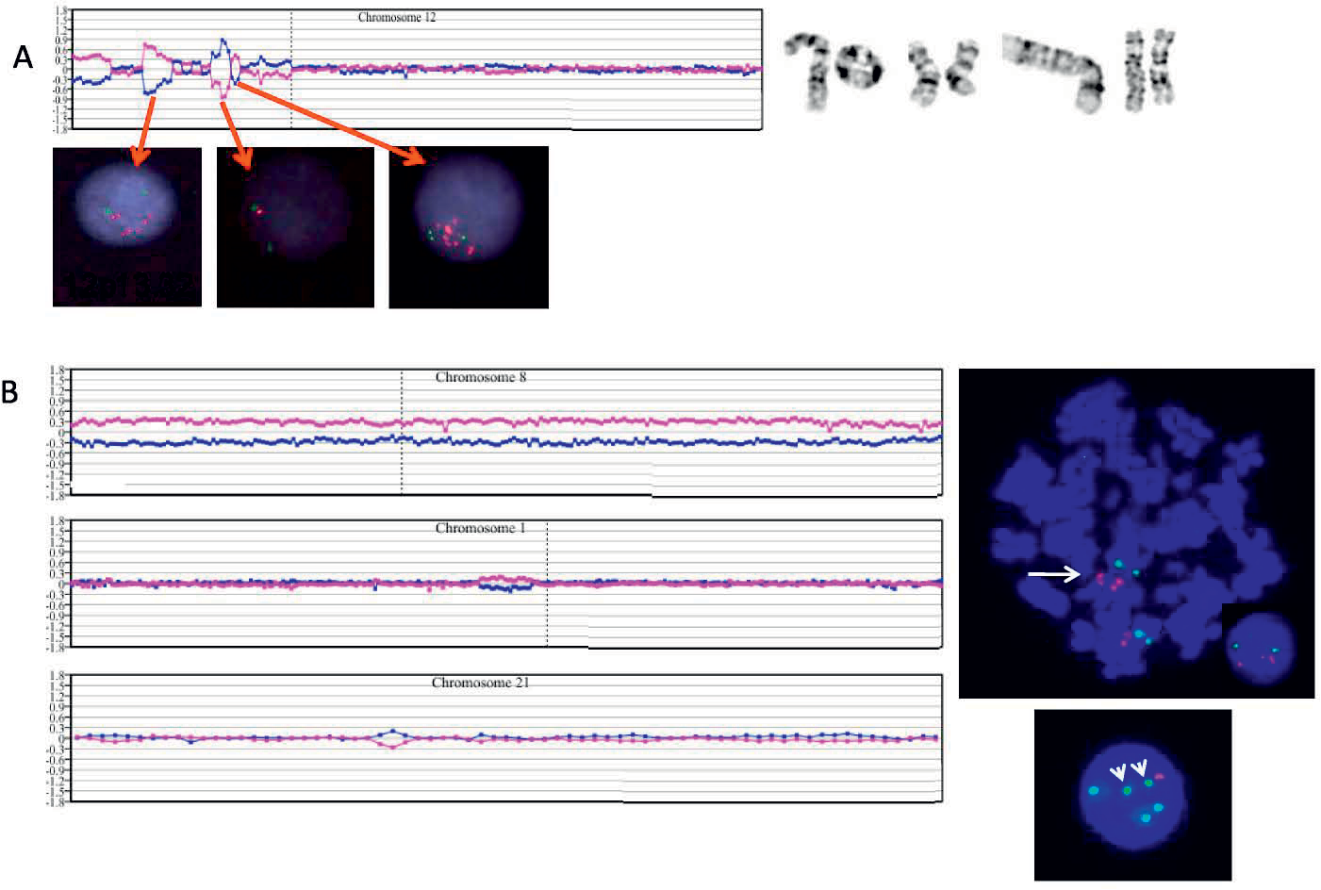
**FISH confirmation studies**. A) Four different chromosome 12 abnormalities in patient #1 and the corresponding aCGH plot. Three duplication/deletion/duplication regions were confirmed by FISH: amplification of CCND2/12p13.32 (RP11-928N17) is denoted by a 7R/2G FISH pattern; deletion at 12p12.3 (1R/2G pattern) confirmed using the RP11-147E12 FISH probe which maps between PLCZ1 and PLEKHA5; and gain of SOX5 (RP11-34a16) at 12p12.1 with a 4~8R/2~ 4G patterns. B) Submicroscopic CNAs in a trisomy 8 patient #21. BAC aCGH plots for chromosomes 8, 1 and 21. Using the dye swap method, the top plot shows trisomy 8 (FISH confirmed in 82.5%), the middle plot shows a gain (duplication) of 1p21.3p12 confirmed by FISH (32%), and the bottom chromosome 21 plot shows a 344-kb RUNX1/21q22.12 deletion. The duplication was confirmed by FISH on metaphase cells using a 1p21 probe (RP11-96F24), which maps within the duplicated segment, labeled in red, and a control probe that maps to 1q24 (RP11-104L21) labeled in green. Arrow indicates tandem 1p duplication in the metaphase cell. The interphase cell shows three red signals and two green signals. Triple-color interphase FISH (lower right) confirms RUNX1 deletion in 10% of trisomy 8-positive cells. The chromosome 8 centromere probe is labeled in aqua (signals not arrowed), a control probe for distal 21q is labeled in green (white arrows), and the 180-kb RUN1 probe (dJ1107L6) is labeled in red. RUNX1 deletion was present in 10% of trisomy 8-posiitve cells.

Likewise, aCGH revealed 23 CNAs in RAEB-2 patient #1 and precisely defined the chromosomes 5, 7, 12, 17, 18, 19 and 20 imbalances. Furthermore, aCGH revealed that two chromosome 5 abnormalities identified by karyotyping, der(5)add(5)(p15.3)del(5)(q34) and monosomy 5, were in actuality three distinct deletions: the der(5)add(5p) was a 5p15.31pter deletion, and a 163-kb 5q22.2/APC deletion, confirmed by I-FISH (73%), and 61.3-Mb deletion of 5q23.1qter also confirmed by I-FISH (69.2%), were present. The dic(6;12) was more complex and involved a marker chromosome resulting in a net loss of 2.6 Mb at 6p25.2. Chromosome 7 showed five discrete non-contiguous regions of deletion by aCGH including two short arm deletions (1.9-Mb deletion of 7p14.1 and 23.12-Mb deletion of 7p13q11.11) and three distinct 7q deletions (1.24-Mb 7q11.22 deletion, 323-kb deletion at 7q21.3, and 62.4-Mb deletion of 7q21.3q36.3). NCAM-specific FISH (60%) confirmed the presence of an unsuspected 49.5-Mb 11q14.1-11q25 gain was a marker chromosome. The 270-kb NF1 deletion and 186-kb 20p deletion were also confirmed by I-FISH (33% and 50%, respectively). Four cytogenetically detected aberrations of the short arm of chromosome 12 [add(12)(p13), inv(12)(p11.2p13), r(12)(p11.2q24.3), and dic(6;12)(p10;q10)] resulted in an equally complex 12p aCGH plot of alternating gains and losses from pter to cen: amplification of CCND2/12p13.32 (7R/2G I-FISH pattern); loss of 12p12.2 (1R/2G pattern) using RP11-147E12, a probe localized between PLCZ1 and PLEKHA5; and gain of SOX5 within 12p12.1 (Figure 1A). Both chromosome 18 aberrations resulted in net imbalances: a 9.7-Mb 18p11.32p11.22 deletion (confirmed by I-FISH, 33%) and a 20-Mb 18q21.32q23 deletion (FISH confirmed 48% with the concurrent control probe localized to a marker chromosome). Similarly, the complex and composite karyotypes of patients #9, #16, and #28 showed unpredictable CNA patterns by aCGH, allowing for more precise definition and mapping of imbalances in samples with considerable chromosome instability.

Hidden CNAs were also observed in non-complex karyotypes (#18, #24, #29). In addition to the 11-Mb deletion of 12q reported in reproducibility study patient #18, an 87-year-old female patient (#24) with "isolated 5q-syndrome", quantified by I-FISH (65.5%), showed an evolving 7.6-Mb deletion of 12p13.1p12.2 by aCGH, confirmed by I-FISH (17%) using the RP11-1069E18/EMP1 probe. A primary myelofibrosis patient (#29) showed a non-complex der(1;7) karyotype; however, aCGH revealed four additional submicroscopic deletions (% of cells showing the deletion by I-FISH in parentheses): a ~300-kb 5q32/TCERG1 deletion (77%); a 380-kb 12p13.1 deletion (~10%); a 1.65-Mb deletion at 13q14.2q14.3 (91%) using a probe for RB1; and a 835-kb NF1/17q11.2 deletion (75%).

**Figure 2:**
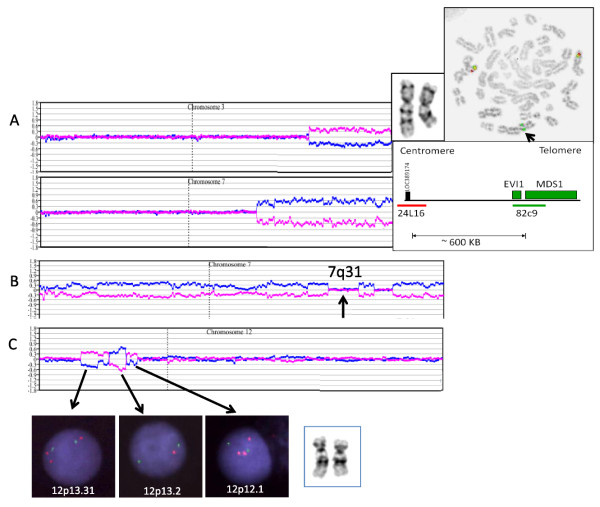
**Unexpected aCGH results**. **(A) **Hidden unbalanced der(7)t(3;7) rearrangement in patient #22. Plots for chromosomes 3 and 7 reveal a 3q gain and 7q loss. Using a homebrew breakapart probe set for EVI1 (bottom right), metaphase FISH confirmed the presence of both EVI1/3q26.2 FISH signals on both chromosome 3 homologues and a single EVI1 (green) telomeric probe to the der(7) chromosome at band 7q21.3 (upper right). This patient also showed a "cryptic" RUNX1 deletion (not shown). **(B) **Non-contiguous CNAs of chromosome 7 resulted in four distinct deletions (one in 7p and three in 7q) in patient #15. The 7 centromere was detected by FISH but is not represented on the BAC array. Using a commercially available 7cen/7q31 FISH probe set, a normal FISH result was reported in this patient because the 7 centromere and 7q31 (arrow) were present in two copies. **(C) **Complex 12p aCGH plot in patient #15 with normal-appearing pair of chromosomes 12. The dup/del/dup 12p CNAs detected by aCGH were not visible by cytogenetics. I-FISH confirmed gain in 12p13.31 with RP11-433J6, deletion at 12p13.2 using RP11-36K5, and duplication of 12p12.1 using RP11-34A16.

Three other MDS patients with non-complex karyotypes showed submicroscopic 344-kb RUNX1 deletions at AML transformation. Trisomy 8 patient #21 (82.5% by I-FISH) showed two accompanying submicroscopic aberrations by aCGH, a duplication of 1p21.3p12 confirmed by I-FISH (32%) and loss of RUNX1 at 21q22.12 confirmed by triple-color I-FISH (~10%) using a small 180-kb RUNX1 probe (dJ1107L6) in trisomy 8 positive cells (Figure 1B). I-FISH using a 500-kb commercially available probe for RUNX1 failed to detect the 344-kb deletion, emphasizing the power of aCGH technology to detect clinically significant submicroscopic abnormalities and the need to use an appropriately sized FISH probe to confirm the smaller submicroscopic CNAs. The second trisomy 8, in patient #26, which was quantified at 22.5% by I-FISH, also showed a suspected RUNX1 deletion at AML transformation. I-FISH using the RP11-77G18 FISH probe that only covered 120 kb of RUNX1 was found to be within background with no additional material remaining to repeat the test using a RUNX1-specific FISH probe. The third patient was a confirmed del(7q) positive t-MDS patient (#22) being evaluated post-treatment for breast cancer. Surprisingly, microarray analysis showed the del(7q) carried an hidden unbalanced der(7)t(3;7) rearrangement with 7q loss and gain of 3q with an EVI1 breakpoint confirmed by FISH (~90%) using two BACs that flank the EVI1 gene at 3q26.2 (Figure 2A). In addition, this patient also showed a "cryptic" RUNX1 deletion (data not shown).

A fibrotic RAEB-1 patient (#15) who failed treatment with lenalidomide and received AZT therapy for 10 years showed a hypodiploid (complex) karyotype with six clonal aberrations. aCGH confirmed the known imbalances, revised multiple breakpoints, revealed small deletions at translocation breakpoint sites, and exposed two complicated rearrangements involving chromosomes 7 and 12. Non-contiguous chromosome 7 resulted in four distinct deletions (one in 7p and three in 7q) (Figure 2B) and as the 7 centromere and band 7q31 were retained, FISH using a commercially available 7cen/7q31 FISH probe set showed a false normal signal pattern. Moreover, the FISH-confirmed dup/del/dup CNAs in 12p were not visible by cytogenetics (Figure 2C). In contrast, loss of CDKN2A, detected by I-FISH (44.5%) only, was not detected by aCGH because probes for 9p21 and this specific gene were not well represented on the BAC array.

The presence of both hypodiploid and hypotetraploid clones in a background of normal cells in two patients (#3 and #16) resulted in a variable log_2 _ratio, confounding data interpretation. Despite the presence of low-level 2n/4n abnormal (three mitotic cells each) clonality in a background of 70% normal cells, the aCGH profile for RCUD patient #3 showed a clear 4.6-Mb deletion at 5q32q33.1, including a hemizygous deletion of RPS14; however, loss of the Y chromosome was not detected because sex chromosome alterations are not obvious using sex-mismatched control DNA and BAC microarrays. Neither aCGH nor FISH analysis detected the 17p deletion. FISH quantitation of 17p deletion was 2% (within background) owing to the low frequency of the near-2n clone (~15%) and a false "normal" FISH pattern in the corresponding 4n clone. Conversely, the near-4n clone in patient #16, estimated at ~10%, showed concordance between the aCGH and cytogenetics results, with two exceptions: chromosome 19 showed greater complexity by aCGH, whereas loss of the Y chromosome was only detected by cytogenetics. Follow-up studies for both patients confirmed the genetic results for both patients.

Clonal evolution of disease was evaluated by aCGH in three patients (#9, #15, and #16). The follow-up samples were compared to their earlier (reference) sample. All three patients showed an increase in blast count with evolution of their complex karyotypes. Clonal evolution was evident by aCGH by the presence of new aberrations, larger or biallelic deletions of pre-existing alterations, or more complex chromosome aCGH plots for a given chromosome (data not shown).

### Limitations of aCGH in a diagnostic setting

Five karyotypically aberrant patients showed normal aCGH results. Patient #5 showed a balanced t(3;12)(p25;q13), and four patients (#4, #10, #25 and #30) showed low-level clonality (>10~15%). Patient #10 received a matched unrelated stem cell transplant for karyotypically normal RAEB in 1996 but relapsed in 2007. The small del(20q) population observed in this patient was within background limits by I-FISH (~2%) and was suspected to be donor-derived based on chimerism studies. The only deletion noted in patient #30 was a 458-kb deletion at 3p12.1 thought to be a CNA of no known pathological significance. This patient had shown low-level der(1;7) clonality, confirmed by I-FISH in ~3-5%, for five years without pathological evidence of disease. Patient #4 showed a del(5q) karyotype, but I-FISH (0.7%) did not confirm a del(5q) in the residual material above background. The sample submitted for patient #25 was aparticulate and hemodilute with only 5% involvement by I-FISH.

The BAC aCGH results detected low-level trisomy 9/del(20q) clonality in a patient with high-grade MDS (RAEB-2 patient #17), quantitated at ~16% involvement by I-FISH, but we found the results were not always reproducible under 20% involvement. In patient #20, the MDS-related del(5q) clone was easily detected by cytogenetics, non-targeted FISH and aCGH; however, this patient also had low-level involvement of hyperdiploid multiple myeloma. The hyperdiploid clone was only detected by plasma cell-specific FISH studies [26], indicating that low-level clonality observed in co-morbid disease states may be masked by the dominant clone, requiring enrichment steps for detection by aCGH.

## Discussion

We undertook this exploratory study to determine if a genome-wide BAC microarray would enhance karyotype precision in MDS/MPN by detecting previously known and clinically relevant submicroscopic CNAs. We also sought to define limitations of array-based karyotyping that might require further evaluation and refinement prior to implementation in the clinical diagnostic setting. These objectives were accomplished by comparing the aCGH results to their corresponding cytogenetics and clinicopathological features. In addition, all new submicroscopic CNAs were verified by FISH analysis. Independent confirmation of unexpected CNAs found by aCGH, using techniques like FISH, multiplex ligation-dependent probe amplification (MLPA), polymerase chain reaction (PCR) or other molecular techniques, including other higher- or lower-resolution microarrays, is critical in a diagnostic laboratory to confirm array results.

Consistent with prior array-based investigations [6,8,27], a number of advantages and challenges for clinical aCGH studies in MDS are evident. Overall, conventional cytogenetics and aCGH show excellent agreement for the commonly observed imbalances found in MDS patients with the added advantage to aCGH of providing more precise details of the alterations including genomic size, gene content and finely mapped breakpoints. As reported by others [8,17,28], the proximal and distal 5q breakpoints were found to be highly variable. Three del(5q) patients presented here illustrate the potential clinical utility of this added information.

Deletions of 5q are observed in ~10-15% of de novo MDS patients; however, only a small subset fulfill the WHO criteria for "isolated del(5q)" syndrome, a haploinsufficiency disorder of the ribosomal protein RPS14 gene associated with low blast counts, a good prognosis and a favorable response to lenalidomide [11,29-31]. The sole del(5q) alteration reported in RARS patient #13 did not include the RPS14 gene and in confirmation, this patient had failed to achieve a response with lenalidomide. Another MDS patient (#24) with "isolated 5q" syndrome showed an emerging submicroscopic secondary alteration. Submicroscopic CNAs have been reported in other "isolated 5q-" patients [17,18]; however, the clinical implications and substratification of added molecular genetics events in these patients are unknown. Nevertheless, the presence of one or more karyotypic aberrations in del(5q) MDS has been associated with significantly shortened overall survival [12,32], suggesting a clinical necessity to monitor the frequency of secondary alterations during treatment. Thirdly, selection of an appropriate FISH probe for monitoring MRD is easily achieved based on aCGH results. For example, the presence of a secondary del(11)(q13q23) aberration in del(5q)-positive patient #11 implied a quantitative MLL/11q23 FISH assessment might have been useful to monitor clone size during treatment; however, FISH studies indicated the MLL gene was neither deleted nor rearranged. Similarly, ETV6 hemizygous deletions are commonly reported in myeloid disorders; however, the del(12p) in patient #23 did not result in an ETV6 deletion but did result in loss of KRAS. Andreasson and colleagues [33] were the first to report that 12p deletions in myeloid disorders do not always involve ETV6, and deletions in this region may occur without visible karyotypic changes, as seen in patient #15. These co-authors further suggest deletion of CDKN1B, a cyclin-dependent kinase inhibitor, or a nearby gene may play a critical role in myeloid malignancies. Selecting a suitable FISH probe from within the aCGH-defined deletion provides a testable clonal marker for a quick quantitative assessment of the subclone at presentation and for MRD testing in follow-up samples, improves laboratory resource utilization and spares the patient the cost of non-informative FISH testing.

In agreement with other MDS investigations using various microarray platforms [6,8,18,19,28], 47% of the MDS samples studied showed hidden CNAs by aCGH. Submicroscopic CNAs were found in both complex and non-complex karyotypes revealing the composition of markers, authenticating questionable calls and improving definition of the imbalances. Hidden complexity frequently involved 5q, 6p, 7q, 12p, and 19p with unsuspected gene amplification exposed in two RAEB-2 patients who quickly progressed to AML. Amplification of CCND2 at 12p13.32 in patient #1 implies disruption of cell cycle progression and loss of hematopoietic regulation [34], whereas amplification of the 19p13.3p13.2 region in patient #16, a 2-Mb region housing many genes, requires further studies to characterize the alteration. The heterogeneity and high degree of chromosome instability observed among the various subtypes of MDS/MPN denotes considerable genomic complexity and underscores the need for a well-designed high-density genome-wide microarray for clinical diagnostics.

The clinical outcome of der(1;7) MDS patients has been a subject of much debate [35,36]. Our study included three patients (#8, #29, #30) with der(1;7)(q10;p10) as the sole aberration based on cytogenetic analysis. Patient 29 presented with marked anemia, thrombocytopenia with dysplastic megakaryocytes and der(1;7)(q10;p10) in 95% of the metaphase cells analyzed. aCGH detected four additional submicroscopic deletions in this patient ranging in size from 310 kb to 1.65 Mb: 5q32/TCERG1, 12p13.1/EMP1, 13q14.2q14.3/RB1, and 17q11.2/NF1. Our aCGH analysis revealed hidden deletions in the RAS regulator NF1 tumor-suppressor gene in two patients. In addition, RAS pathway mutations with and without RUNX1 alterations have been reported in -7/7q- alterations in AML arising from MDS and chronic myelomonocytic leukemia with myelodysplastic features [37-39]. Clinical trials designed to evaluate the ability of RAS inhibitors in MDS have shown limited success, hinting that further patient stratification may be needed to develop RAS inhibitors and mTOR-directed therapeutics [40]. The second der(1;7) patient (#8), classified as RCMD with a history of aplastic anemia, showed a suspicious 13q deletion by aCGH, as an evolving secondary alteration. The third patient (#30) showed low-level der(1;7) clonality for five years with no pathological evidence of disease and a normal aCGH result. These data suggest the presence of submicroscopic CNAs in der(1;7)-positive patients may explain the morphologic and clinical heterogeneity observed in myeloid disorders harboring this specific chromosome aberration.

MDS patients face a ~25% risk that the disease will transform into AML. Cooperating genetic events between two mutation classes appear to play a role in leukemogenesis, namely, inactivation of a hematopoietic transcription factor resulting in loss of cellular differentiation, along with activation of the tyrosine kinase RAS-BRAF signal transduction pathway to stimulate cell cycling and proliferation [37]. In support of this hypothesis, cryptic CNAs were detected in three high-risk MDS patients with non-complex studies at AML transformation. Two isolated trisomy 8 cases (#21, #26) in this study showed a ~340-kb deletion of RUNX1 corroborating the findings of cryptic aberrations in four of 10 trisomy 8 MDS patients reported by Paulsson and co-authors [16]. The third patient (#22), with a submicroscopic deletion of RUNX1, showed a hidden unbalanced EVI1/3q26.2 translocation masked as a 7q deletion, which leads to EVI1 overexpression [41]. Because RUNX1 and EVI1 mutations are frequently reported in high-grade MDS at transformation to AML [37], and EVI1 overexpression is a poor prognostic and risk stratification marker in AML [41,42], we agree with Gondek and colleagues [5,6] that aCGH will improve patient management and prognostication in MDS.

Balanced rearrangements cannot be detected by aCGH. The frequency of balanced translocations in MDS is estimated to be less than 10% with most translocations involving MLL, EVI1 and DEK/NUP214 [21], which suggests that conventional cytogenetics and aCGH are complementary assays in the clinical workup of the myeloid disorders. However, a recent modification of aCGH known as translocation-CGH may provide a new approach to detect prognostically important balanced translocations in neoplastic disorders in the near future [43].

A second limitation of aCGH in diagnostic settings is the inability to detect imbalances reproducibly when the tumor burden is below 20% [5,6,44]. Problems detecting low-level clonality were observed in four different clinical situations: 1) a normal aCGH result in a patient with a karyotypic-aberrant clone in the absence of morphologic disease; 2) low-level clonality in low-grade MDS, in particular with del(20q) or loss/rearrangement of a sex chromosome; 3) detection of the dominant clone only in a co-morbid patient; and 4) difficulty interpreting emerging subclones associated with clonal evolution of disease. Co-morbidity and loss of the Y chromosome occur most frequently in older patients. Whereas low-level loss of the Y chromosome is typically considered an age-related phenomenon without pathological consequences, other sex chromosome aberrations in MDS, such as the idic(Xq), are important to report.

The detection of emerging clones by aCGH for evaluation of clonal evolution of disease was also challenging. Because MDS samples are heterogeneous with multiple cell types and varying degrees of cellular differentiation, enrichment techniques using highly purified CD34+ positive progenitor cells may result in improved microarray resolution Moreover, aCGH should not be used as an MRD assay, however, the detection of pathogenetic CNAs at presentation allows for disease-specific FISH and PCR MDR assays at follow-up.

Two additional factors complicating data interpretation were variability of the log_2 _ratio in samples with multiple clones, especially cases with 2n and 4n clones, and distinguishing true pathogenetic genomic alterations from benign CNAs. In the first instance, interpreting aCGH results became unclear when the frequency of one of the 2n/4n subclones rose above 10%. In this situation, FISH results are also misleading because a monosomy in a near-diploid clone will show a "false normal" signal pattern in the 4n clone. Lastly, ~40 benign CNAs were observed in this study, with most found within segmental duplications. Defining true tumor-associated genomic alterations from non-pathogenetic CNAs requires experience, access to large public databases of normal variants, and perhaps in some neoplastic disorders, comparison of normal and tumor tissue in the same patient.

Our exploratory results using a genome-wide BAC microarray provide preliminary evidence that chromosomal microarray testing holds great promise for augmenting conventional cytogenetics in MDS/MPN. These results also underscore the importance of continued efforts to improve DNA microarray technology in oncology practice. Because this was a proof-of-principle study, our BAC platform did not have proper coverage of the hematopoietic transcription factors and genes associated with the RAS-BRAF signal transcription pathway; in particular, poor probe coverage at 9p21 resulted in a missed CDKN2A/CDKN2B deletion. To facilitate the implementation of clinically relevant genomic information in MDS/MPD, a genome-wide, high-resolution microarray targeting disease-relevant candidate genes, e.g., TET2, IER3, TIRAP, CBL and ASXL1 [18,45-48] and regions of known chromosome instability is recommended. The decision to use an oligonucleotide-based, SNP-based or an oligonucleotide/SNP hybrid platform must be evaluated for test accuracy, validity and clinical relevance within a clinical trial setting.

## Competing interests

BB, RS, and LGS are currently employed at Signature Genomic Laboratories, a subsidiary of PerkinElmer.

## Authors' contributions

MLS was the principal investigator, wrote the paper and takes primary responsibility for the paper. MO and SJF provided the test samples and clinical information. KG performed the pathology review. VB and YH performed the laboratory work for this study. DDS participated in the statistical analysis. MLS, BB, and LGS co-ordinated the research. LM and RS were involved in the discussions. All authors have read and approved the final manuscript.
